# Supplementary material for: Multi-stakeholder perspectives on gender-based violence in marital relationships: A qualitative study in rural India
Source: PLOS Glob Public Health. 2026 May 8;6(5):e0006364. doi: 10.1371/journal.pgph.0006364 (PMC13155619; doi:10.1371/journal.pgph.0006364)
Supplement: Attachment — Submitted filename: Response to Reviewers.docx [file pgph.0006364.s001.docx]

**Responses to Reviewers**

**Multi-stakeholder Perspectives on Gender-based Violence in Marital Relationships: A Qualitative Study in Rural India.**

**Note:** The lines mentioned below correspond to the line numbers in the clean version of the manuscript.

**Reviewer #1**

I appreciate the authors for conducting this much needed public health research. Minor revisions need to be done before next stage.

**Comment 1.** Abstract: kindly write a structured abstract with more details on research results, conclusion and recommendations.

**Response:** Thank you for the comment. Abstract has been revised (lines 20-37). The revised abstract is as below.

In India, an estimated 41% of women report experiencing domestic violence in their lifetime and up to 28% report violence during pregnancy. Our study aims to understand local perceptions/attitudes and lived experiences of GBV victimisation and perpetration among married couples in rural Rajasthan. We conducted a focus group discussion with community health workers (n=7) and interviews with young married men (n=17) and women (n= 21), and local government officials (n=2). The data was analysed using thematic analysis with triangulation conducted across methods and researchers.

Our findings reveal that women experience multiform violence, including physical, sexual, and economic violence. In the continuum of GBV, violence stems from everyday conditions of disempowerment produced through early marriage and reproduction, norms of hegemonic masculinity, and community norms that normalise violence against women. These conditions of disempowerment lead to women’s isolation through control of mobility and surveillance through technology, setting the stage for further violence to occur. Violence prevention efforts in the region must follow a multi-level approach to reduce child marriage through community education and improved enforcement of legislation, enhance reporting and identification alongside help-seeking behaviours through formal or informal channels, transform rigid gender norms, and leverage key actors in the family system such as the in-laws or matchmakers as potential agents of change.

**Comment 2.** Key words: add substance use, masculinity, IPV, GBV, alcoholism, marital rape

**Response:** These have been added. Kindly see revised keywords below (line 38-39).

Keywords: women’s health, violence, gender, marriage, mental health, substance use, masculinity, IPV, GBV, alcoholism, marital rape

**Comment 3.** Introduction:

Line no 35: instead of underage marriage use the word child marriage and mention child marriage act and POCSO act as well

**Response:** Thank you for the comment. Line no. 44 has been revised as follows: Further, despite laws prohibiting child marriage, India has a high proportion of child marriage(2,3).

**Comment 4.** Line no 36: mention latest NFHS results

**Response:** Thank you for the comment. Lines 44-46 are revised as follows: According to the National Family Health Survey- 5 (2019-2021), 38% of women between the ages of 20-49 years report getting married before they turn 18(1).

Latest statistics also added in lines 129-130: In Rajasthan, 24% of ever-married women aged 18-49 have experienced physical violence and/or sexual violence and violence is most commonly perpetrated by husbands(1); and lines 122-124: Rajasthan is an economically disadvantaged state wherein 28.3% of rural women between the ages 20-24 are married by the age of 18 years, and 4.2% rural girls between the ages of 15-19 are mothers or pregnant(1).


**Comment 5.** Line no 44-49: add dowry harassment, dowry death, honour killing and bride burning data

**Response:** Thank you for the comment. Section has been revised and data added for honour killing, dowry deaths, bride burning. See lines 59-68 revised as follows:

In India, an estimated 29% of women aged 18-49 have experienced physical violence since the age of 15 and 32% of ever married women aged 18-49 have experienced spousal violence(1). In 2023, 38 cases of honour killings were reported, though sources indicate that honour-based killings are largely under-reported(12,13). Dowry-related violence is also commonly implicated. According to the National Crime Records Bureau (NCRB), a staggering 6156 dowry deaths cases were reported to the police in 2023. The majority of dowry-related deaths are due to ‘bride burning’ where a woman is doused with flammable liquid and set on fire(14). The reported rates of cruelty by a husband or his relatives rose by 53%, from 18.5% in 2001 to 28.3% in 2018 per 100,000 women between the ages 15–49 years(15,16). Further, in 2023, a total of 29,670 rape cases were reported, with 852 rape victims reported under the age of 18(16).

**Comment 6.** Add existing dowry harassment act, domestic violence act, of relevant mental health care act

**Response:** Thank you for the helpful suggestion. Kindly see lines 68-74 revised as follows:

Despite the existence of several legislative acts such as The Protection of Women from Domestic Violence Act, 2005(17), Dowry Prohibition Act, 1961 (18); and The Protection of Children from Sexual Offences (POCSO) Act, 2012 (3), gender-based violence remains pervasive. National statistics are likely to be underestimates due to underreporting caused by stigmatisation of help-seeking for domestic violence, lack of standardised measurements, and inadequate service provider responses(7) .

**Comment 7.** 103-110- what was the formal training provided to Sathins add details and references

**Response:** Thank you for the helpful comment. Kindly see lines 152-155 revised as follows:

*Sathins* are trained on details of women-centric schemes such as Mahila Shakti Kendra; community outreach and leadership; legal protections and rights and opportunities for women; and improving dialogue and collaboration between women groups.

**Comment 8.** Any vicarious trauma? How was it prevented?

**Response:** Thank you for the question. Please see lines 219-221 as revised below: *To prevent vicarious trauma among research team members, daily debriefs were conducted to focus on reflections and share resources for self-care.*

**Comment 9.** 248-249: need to reflect whether this statement actually reflects the restriction of woman’s autonomy

**Response:** These lines do not correspond to a section on agency but is instead a quote: *Violence shouldn’t happen. It is wrong. Girls go away with boys; mistakes [sexual coercion] happen to girls at an early age. (Community health worker)*

Kindly let us know what this refers to so we can make the necessary changes.

**Comment 10.** Discussion: authors need to strengthen discussion using intersectional feminism lens to write about the plight of married women in India

**Response:** We thank the reviewer for this important suggestion. We have made several changes to the manuscript to include perspectives on caste, systemic dynamics, kinship, and hegemonic norms of masculinity among others. The excerpts from these lines are as follows:

Lines 588-605:

Gender-based violence in rural Rajasthan operates along a continuum—from social conditions such as hegemonic norms of masculinity, constrained agency, and expectations around marriage, to mechanisms of discipline and control including social isolation and monitoring, and finally to overt forms of physical, sexual, and emotional violence. These findings underscore the need to understand GBV as embedded within broader patriarchal kinship structures that shape gendered expectations, authority, and control within marriage, and suggest that effective interventions must engage with these relational and multi-level dynamics.

The findings suggest that these systemic dynamics are deeply shaped by kinship ideologies that organize expectations of marriage, duty, and relational roles(11). These ideologies produced similarities in how men and women understood and justified violence within marital relationships. At the same time, women were not uniformly passive, and instances of negotiation, reinterpretation, and subtle forms of resistance were evident in their accounts. However, the broad convergence in perspectives across genders underscores the strength of hegemonic patriarchal norms in shaping what is seen as acceptable or inevitable within marriage. This pattern resonates with ethnographic work from North India that shows how gendered norms are internalized and reproduced across social actors, even while leaving room for alternative moral, expressive spaces and agency(33–35).

Lines 613-618

At the same time, ethnographic work from North India highlights that women’s voices are not entirely silenced, but may be expressed in alternative or less visible spaces, including through collective action, narrative, and everyday forms of negotiation(37). However, the responses in this study—elicited in formal interview settings—largely reflected dominant and publicly sanctioned norms, underscoring the strength of hegemonic expectations.

Lines 622-626

The problematic nature of such beliefs shows how violence becomes socially sanctioned when women deviate from expected gender norms, particularly those governing obedience, mobility, and domestic roles. The irony, however, is that women who are less empowered, economically and educationally, are also at high risk of experiencing violence(40).

Future GBV programming must situate the changing landscape of women’s empowerment in India within the intersectionality of class, caste and gender, considering that the findings reveal that one size does not fit all. These findings must be situated within the intersection of caste, class, and gender in rural Rajasthan, where hierarchies of labour, land ownership, and social status shape both exposure to violence and access to support. While this study was not designed to systematically compare caste groups, the patterns observed suggest that marginalised communities may experience compounded vulnerabilities linked to economic precarity and social exclusion(28,41). Further ethnographic work is needed to more fully understand how these dynamics vary across caste and community contexts.

At the same time, the regulation of women’s sexuality and mobility observed in this study resonates with broader literature on honour (izzat) and sexual purity in North India. Some scholars have argued that processes of social mobility and modernity are associated with the adoption or reinforcement of more restrictive, brahmanical norms governing women’s sexuality across caste group(33–35). However, the specific context of Sri Ganganagar—characterized by a relatively recent settlement history and a demographic composition in which dominant caste groups are not uniformly upper-caste Hindu—suggests that these dynamics may not map neatly onto classical formulations of brahmanical patriarchy. Rather, concerns around family honour and women’s conduct appear to be locally configured, while still producing similar constraints on women’s autonomy.

Lines 663-672
These findings also point to the ways in which patriarchal kinship systems place normative pressures on men, particularly in relation to provision and authority within the household. When these expectations cannot be met, they may generate frustration and contribute to patterns of alcohol use and violence. This underscores that patriarchal systems not only constrain women but also shape men’s experiences in ways that may perpetuate cycles of harm(45).

The strength of these kinship ideologies was evident in the normalisation of violence within marriage, which was widely described as an expected part of married life and one for which long-term support does not exist.

Lines 708-715

There are several research, political, and policy implications of our findings. These implications must be understood within the specific context of rural districts such as Sri Ganganagar, where geographic distance, agricultural labour relations, and reliance on institutions such as the family (mediated through bicholas or marriage brokers) and panchayats, both formal and informal, shape both the experience of violence and the possibilities for intervention. These contextual features distinguish rural settings from the predominantly urban contexts in which much GBV research and programming has been developed, where service access, resources, mobility, and institutional engagement may differ significantly.

**Comment 11.** Health system strengthening: one stop centres are actually a failure model. Please critically evaluate this recommendation and suggest feasible scalable culturally appropriate recommendations such as capacity building of ASHAs, AWWs, ANMs, CHOs. Why AB HWCs, PHC, AWC cannot act as safe spaces for women?

**Response:** We thank the reviewer for this important comment. Based on our assessment of the existing One Stop Centre in the region, we would like to highlight that while the centre had flaws, it was functioning well and was well-staffed with women being well-served. We have highlighted this in the discussion section 695-697

At the same time, field observations by members of the research team (AB) indicated that some institutional responses, such as the One Stop Centre in the study area, were functioning and accessed by women, with the availability of counselling, legal, and police support. This suggests that while structural and social barriers to help-seeking remain significant, existing services may offer important points of support when accessible and trusted. **Comment 12.** VHSNCs - why can’t they advocate for the rights of women, what are the opportunities

**Response:** We thank the reviewer for this important suggestion. The role of Village Health, Sanitation and Nutrition Committees (VHSNCs) in advocating for women’s rights and addressing GBV was not directly explored in this study.

However, our findings point to several contextual factors that may shape the potential of such community-based platforms. These include the normalization of violence within marital relationships, the framing of intimate partner violence as a “private matter,” and the broader patriarchal and kinship structures that influence community norms and decision-making. In such settings, community-level institutions may themselves be embedded within these same social dynamics, which could limit their ability to effectively advocate on issues of GBV.

At the same time, we agree that VHSNCs represent a potential opportunity for intervention. Strengthening their capacity through training, sensitization, and clearer mandates may enable them to play a more active role in community-level responses to GBV, particularly if aligned with broader efforts to engage families and local governance structures.

**Comment 13.** You can also elaborate on challenges faced by ministry of WCD in curbing child marriages and implementing Beti Bachao Beti Padhao campaign strategies

Thank you for the helpful suggestion. Kindly see revised lines 725-733 as follows:

A key challenge in child marriage prevention is in part caused by the legal design where child marriage is legally recognised per se, unless nullified by either of the parties involved(52). To counter this, National Commission of Women proposed The Draft Compulsory Registration of Marriage Bill, though it continues to be in between legislative proceedings and has not been implemented yet(53,54). There is a need for compulsory age verification at the time of marriage and combat the pervasive use of fake birth certificates(53). National campaigns such as “Beti Bachao, Beti Padhao” (Save Girls, Educate Girls) have led to positive outcomes over time in Haryana, Rajasthan, and tribal regions of Jharkhand, key issues persist in changing deep-rooted patriarchal beliefs and maintaining adequate monitoring(55).

**Reviewer #2 

Comment 14.** The paper is well written. The study provides valuable data in an understudied setting. It will benefit from some small tweaks for increased clarity- In the introduction, the denominator is unclear when you are citing NCB statistics. Please clarify.

**Response:** Thank you for the helpful comment. This has been corrected in the revised lines 64-68 as below:

The majority of dowry-related deaths are due to ‘bride burning’ where a woman is doused with flammable liquid and set on fire(14). The reported rates of cruelty by a husband or his relatives rose by 53%, from 18.5% in 2001 to 28.3% in 2018 per 100,000 women between the ages 15–49 years(15,16). Further, in 2023, a total of 29,670 rape cases were reported, with 852 rape victims reported under the age of 18(16).

**Comment 15.** The methodology is sound. What was the purpose of the double blind though, as it wasn’t an experimental study? How may hiding the purpose of the study have affected results? It might also be useful to provide some information about your SSI and FGD questions to ensure transparency.

**Response:** Our study was not a double-blind study. Participants and researchers had complete and full information about the study outcomes, methods, and purpose. Our methods section also reflects the same. The issue of double-blind may have arisen from the wording around how we developed dummy questionnaires and a backstory for researchers to reduce risk of any escalation of violence, in case a perpetrator husband or family member, walked into the interview, or found the resource of helpline numbers that we shared with the participants. This was done based on recommendations from “Researching Violence Against Women: A Practical Guide for Researchers and Activists” by Lori Heise and Mary Ellsberg. We have now reworded this section of the methods (lines 207-218) to clarify this further. Let us know if this works. Please see excerpt below.

We developed a number of tools and procedures, informed by previous practice, to safeguard participants from potential risks if household or community members were to find that the study centrally focuses on gender-based violence, which could lead to conflict or violence. These included developing a backstory for researcher and creating a dummy questionnaire with questions unrelated to GBV and DV, in the case that a spouse or a family member who is a perpetrator of violence, walks into the interview; and identifying private spaces outside the household to conduct SSIs and the FGD. The dummy questionnaire was not used as a data collection tool – it served as a risk mitigation strategy(32).

**Comment 16.** Also, what is the definition of violence you are using? does it encompass all kinds of physical, emotional, sexual and financial abuse?

**Response:** Thank you for the helpful suggestion. We have added our definition of GBV. Kindly see revised lines 106-110 as below:

We defined GBV as “any act of gender-based violence that results in, or is likely to result in, physical, sexual, or mental harm or suffering to women, including threats of such acts, coercion or arbitrary deprivation of liberty, whether occurring in public or in private life” (25). Our definition, thereby, captured all forms of violence including physical, sexual, economic, and emotional violence perpetrated by married partners.

**Comment 17.** I think the theme code ‘marriage as a single blind experiment’ needs to be rephrased. This code, while witty, does not accurately capture what is essential deception and lack of agency for women when making a choice of their partner. An experiment has a purpose- what is it in this case?

**Response:** Thank you for flagging this. Please see revised as “marriage as an entry into the unknown” in lines 270.

**Comment 18.** Some of the sub themes can be explained, in a table if needed, for clarity.

**Response:** Thank you for the helpful suggestion. We have added a second table to the manuscript (Table 2) that lists each of the themes and subthemes across the three categories for better readability and clarity. Kindly see line 241.

**Comment 19.** While data triangulation is helpful, in this instance, it might be helpful to also establish differences in the ways in which violence is viewed across genders.

Thank you for this helpful suggestion. We have made the distinction across young men and women in several places across the results section of the manuscript.

Kindly see revisions in the following lines:

Lines 251-263:

Most men respondents stated that women are expected to be solely responsible for household work and reproduction. Similarly, many women expressed that fulfilling these normative expectations was their responsibility. At the same time, women often revealed dissatisfactions with their life after marriage and the burden of chores being a source of family conflict. One woman stated, “*Earlier, there were not many responsibilities. There is no issue if we do or don’t work at parent’s home, but here, we have to do it. There is also the responsibility of the children now. Many changes occur after the marriage. Life before marriage with parents is very good while it is not that good after marriage.*" Many female participants reported having to learn marital roles and responsibilities at a very young age (as young as 15), including feeling obligated to have sex with their husbands. Fewer men described feeling unhappy with an early marriage. One man stated, “*I wanted to have a job first. After marriage, it becomes more restricted; you can’t go anywhere, (you) can’t do what you want to do.*”

Lines 282-293

Most married men and almost half as many married women endorsed similar beliefs that women should “obey” the husband and maintain the honour (izzat) of the family by demonstrating modest behaviour in terms of clothing (including veiling) and restricted social interactions. One woman stated, “*She (a woman experiencing violence) must say to him (husband) that I will listen to you and obey whatever you will say. If she will argue about right and wrong with him, then you can understand the outcome of such an argument.*” As such, there was an agreement between men and women respondents that any deviation from these norms was said to warrant “discipline and control” towards the woman. Further, men reported that violence in such situations was ‘justified’ in order to keep the woman ‘beneath her husband’. Violence however was also reported when women met the normative expectations. One woman stated, “*Although I used to wear a veil, he was habitual of beating me. “My wife should not see anyone, should not talk to anyone.*”

Lines 307-309

Young men, women, and community health workers stated in agreement that the woman’s expected role in the marriage is to keep the family together, even in situations of conflict or violence.

Lines 329-332

Many male respondents reported feeling entitled to sex, highlighting broader community perceptions. One man stated, “*Look, the main thing in life is sex. You understood, right? I continue to have sexual desire fulfilled and I continue to work well also. Now, I don’t have any tension, no matter whatever I do. And my wife is also very nice; she is not very demanding. She doesn’t say get me this, get me that, she doesn’t say all that.*"

Lines 359-363

While a preference for sons persisted, an emerging pattern suggested that daughters were desired and even better treated if the woman had already had a son. For instance, one woman stated her sister-in-law, *“I have a son, now (God) give me a girl, then I will get the operation (sterilization).”* Similarly, another woman spoke of her husband, *“No, he (husband) never made any differences in both of them (son and daughter). He loves them very much.*

Lines 477-479

Control and isolation also occurred when the husband feared infidelity or had feelings of jealousy. Most young women reported that such fears or feelings of jealousy were unfounded; however, their attempts to prove otherwise did not de-escalate violence in most situations.

Lines 508-511

Most men did not report an understanding of why women silence themselves or do not seek help. A young man stated, “*But in our village here, the husbands injure their wife’s heads completely, and despite of that, they don’t go the police station also. They say that he is my husband. I fail to understand why that is so.*"

Lines 529-532

One woman stated, “*We, the girls of poor families, think like, if we will go and tell our mother (about the violence), still the problem will not decline, it will only progress further. What is the benefit if such matter progresses further? Ultimately, I will need to live there only (at the in-laws).”*

Lines 563-565

Young men emphasised that a woman experiencing violence by their husbands should leave the marriage but offered limited insight into barriers for help-seeking and implications for women.

**Comment 20.** While you have representation across class and caste, it might be useful to comment on whether your data revealed trends based on intersectional parameters. For instance, did caste disenfranchisement affect norms of masculinity? Did Brahmanical patriarchy related values affect notions of sexual purity?

We thank the reviewer for this helpful suggestion. We have added intersectional parameters across the discussion section on how gender-based violence is linked to Brahminical patriarchy. As our study did not elicit detailed responses on how caste intersections with notions of sexual purity or masculinity, we have positioned these arguments in the discussion section of the manuscript, as opposed to trends released from our study findings. Kindly see the revised lines as below (638-647):

At the same time, the regulation of women’s sexuality and mobility observed in this study resonates with broader literature on honour (izzat) and sexual purity in North India. Some scholars have argued that processes of social mobility and modernity are associated with the adoption or reinforcement of more restrictive, brahmanical norms governing women’s sexuality across caste group(33–35). However, the specific context of Sri Ganganagar—characterized by a relatively recent settlement history and a demographic composition in which dominant caste groups are not uniformly upper-caste Hindu—suggests that these dynamics may not map neatly onto classical formulations of brahmanical patriarchy. Rather, concerns around family honour and women’s conduct appear to be locally configured, while still producing similar constraints on women’s autonomy.

**Comment 21.** Please locate your results in the context of existing research on GBV primarily conducted in urban areas. Are there context-specific differences you noted? Also comment briefly on resources available for help, in lieu of PWDA act or lack of support for marital rape, and the reality of how these operate in the rural context, which helps to put some of the help seeking limitations and gaps into perspective.

We thank the reviewer for this helpful suggestion. We have added a section in the manuscript to address how the study findings must be situated within the rural context of Sri Ganganagar; and what distinguishes such rural settings from urban contexts where most GBV research is developed.

Please see the revision in lines 708-715 as below:

There are several research, political, and policy implications of our findings. These implications must be understood within the specific context of rural districts such as Sri Ganganagar, where geographic distance, agricultural labour relations, and reliance on institutions such as the family (mediated through bicholas or marriage brokers) and panchayats, both formal and informal, shape both the experience of violence and the possibilities for intervention. These contextual features distinguish rural settings from the predominantly urban contexts in which much GBV research and programming has been developed, where service access, resources, mobility, and institutional engagement may differ significantly.

Thank you for the helpful suggestion on the lack of support for marital rape and how it limits help seeking. We have revised the discussion section to address this. Please see lines 672-678 revised as below:

Sexual violence or marital rape in particular, was highly normalised. India is one of the 36 countries where marital rape is not a crime(46). The Domestic Violence Act recognises sexual violence within marriage, but does not criminalise it, and instead offers civil remedies such as separation and divorce(17). Divorce or separation, however, is uncommon. Our findings are in line with research from South Asia which shows that divorce is often not sought because birth family support may not exist or the option of earning income is not available(47).

**Comment 22.** It might also be helpful to situate the data using existing theories in India, such as local masculinity conceptualisations, notions of collectivist of honour, or the Chakrabhed cycle of marital and natal family violence by Gupte and More in Padma Deosthali’s 2012 book.

We thank the reviewer for this helpful suggestion. We agree that the concept of cyclical patterns of violence across natal and marital households is an important contribution to understanding women’s experiences of GBV in India. While we were not able to locate the specific referenced chapter on the “Chakrabhedh cycle” in Deosthali (2012), we have incorporated this broader insight into our discussion by highlighting how violence is normalised within marital relationships and shaped by kinship structures that extend beyond the immediate household, including the roles of family-mediated arrangements (e.g., bicholas) and community institutions such as panchayats. We have also strengthened the discussion of kinship ideologies and their role in structuring women’s experiences of violence, which we believe captures the underlying dynamics referenced in this suggestion.

**Note from authors:**

In addition to the revisions outlined above, we would like to notify the reviewers of a correction in the socio-demographic table (Table 1). In the previous version of the manuscript, the caste group “Jatt Sikh” was misclassified as General Caste. While Jatt Sikhs are categorized under the General Caste in most Indian states, they are classified under the Other Backward Classes (OBC) category in Rajasthan. This error has now been corrected, and the revised figures are reflected in Table 1.
